# Global conservation of phylogenetic diversity captures more than just functional diversity

**DOI:** 10.1038/s41467-019-08600-8

**Published:** 2019-02-20

**Authors:** Nisha R. Owen, Rikki Gumbs, Claudia L. Gray, Daniel P. Faith

**Affiliations:** 1 EDGE of Existence Programme, Zoological Society of London, Regent’s Park, London, NW1 4RY United Kingdom; 2 On the EDGE Conservation, London, W2 5EU United Kingdom; 3 Science and Solutions for a Changing Planet DTP, Imperial College London, London, SW7 2AZ United Kingdom; 4 The Australian Museum Research Institute, The Australian Museum, Sydney, 2010 Australia

**Arising From** F. Mazel et al. *Nature Communications* 10.1038/s41467-018-05126-3 (2018)

The biodiversity measure, phylogenetic diversity (PD), links evolutionary history to the conservation of feature-diversity (broadly, the different evolutionary features of species), and so to future options for humanity (option value)^1^. Mazel et al.^2^ claim that (1) PD must perform better than random in capturing functional diversity (FD) if it is to have any validity for conservation; (2) PD captures FD unreliably; and (3) we may need to abandon the use of PD in conservation, depending on the outcome of further FD randomisation tests. We argue that Mazel et al.^2^ misrepresent the PD/feature-diversity framework as restricted to functional traits, and we illustrate how a conservation focus on functional traits could lead to the global loss of PD, feature-diversity and option values. The core rationale for PD conservation initiatives, such as the EDGE of Existence programme, should continue to build on the link from PD to broad feature-diversity^1,3,4^, not a link to a few nominated functional traits.

Mazel et al.^2^ explicitly point to Faith’s^1^ original broad feature-diversity arguments in describing the idea that conserving PD will conserve a wide variety of forms and functions. However, Mazel et al.^2^ then adopt a narrower functional perspective, asserting that such diversity can be measured as FD, calculated using selected traits of assumed ecological relevance (e.g., four mammalian traits: diet, body mass, activity cycle, foraging height). Mazel et al.^2^ incorrectly synonymise FD with feature-diversity by misrepresenting Faith’s reference to feature-diversity^1^ as a reference to FD, and Faith’s reference to future options arising from feature-diversity^1^ as a reference to future options from FD.

This misrepresentation underpins their false claim: “the fundamental phylogenetic gambit at the heart of all PD-based conservation strategies …[is that]… maximizing PD captures more FD than randomly choosing species.” Because they incorrectly equate PD’s broad feature-diversity with their narrowly defined FD, Mazel et al.^2^ have no justification for this claim that failure to recover FD in their randomisation tests casts doubt on all PD conservation initiatives.

One such PD conservation initiative, the EDGE of Existence programme^5^, is characterised by Mazel et al.^2^ as following the logic of their FD phylogenetic gambit. In reality, an original rationale for EDGE explicitly drew on Faith’s^1^ feature-diversity arguments: Isaac et al.^5^ noted that “phylogenetic diversity is clearly related to character diversity”, arguing that PD-based scores indicate unique features and potential future utilitarian value. This general feature-diversity/future options link has supported the prioritisation of EDGE species for conservation, including those that might otherwise be neglected. The EDGE rationale echoes the history of studies on how phylogeny is informative about both known and unknown features of organisms^3,6^. Early studies examining character/feature data and phylogenies^3,4^ also provided tests corroborating the specific rationale for PD—that shared ancestry can generally account for shared features among species. Mazel et al.’s^2^ test of their FD gambit does not assess whether PD captures a wide range of features; indeed, a perfect PD-feature relationship can produce a failure in their test^7^.

The well-corroborated PD-features relationship^3,4^ has supported the use of EDGE information by the Intergovernmental Science-Policy Platform on Biodiversity and Ecosystem Services (IPBES), as one indicator for “maintenance of options” (one of “nature’s contributions to people”)^8^. IPBES reports global estimates of total imperilled PD, over six major taxonomic groups^8^. The IPBES Asia-Pacific assessment^8^ provides recent examples of surprising global benefits that have been discovered, illustrating the broad range of these often-unfamiliar evolutionary features. These include the discovery that funnel-web spider venom (*Hadronyche infensa*) is the unlikely source for medication to avoid brain damage caused by strokes^9^, and that a substance in Tasmanian Devil milk (*Sarcophilus harrisii*) fights antibiotic-resistant bacteria^10^. This unusual mammal feature illustrates the scope of feature-diversity, in contrast to Mazel et al.’s^2^ limited four ecological traits. Other examples highlight how the feature of interest originated in an ancestral lineage (e.g. medicinal features in plants^11^), further corroborating the PD rationale.

The EDGE of Existence programme interprets PD and feature-diversity as indicating multiple values of biodiversity. A species’ unique contribution to PD (Evolutionary Distinctiveness^5^) not only indicates its contribution to option value, but is a proxy for its many unique evolutionary novelties – a heritage that we marvel at (and link to bequest and existence values). EDGE also has acknowledged the scope for such PD conservation to preserve functional traits^12^. Such inclusivity is sensible, and we welcome further exploration of the PD and feature-diversity link, including FD. The weakness of Mazel et al.’s^2^ study is the unwarranted restriction that the only rationale for PD conservation is their FD gambit.

Reflecting their exclusive FD focus on ecosystem functions and services, Mazel et al.^2^ argue that conservation increasingly focuses on local scales, suggesting that if their gambit were valid, PD could prioritise species in “conservation programs such as the EDGE of Existence who might be planning their list of species to protect on a coral reef”^13^. However, the EDGE of Existence programme actually considers global-scale PD conservation and associated future options, and does not set priorities locally (such as within individual coral reefs). In a simple example (Fig. 1) we show that in such a local-PD prioritisation, some EDGE species may never become a priority, resulting in their global loss.

**Fig. 1:**
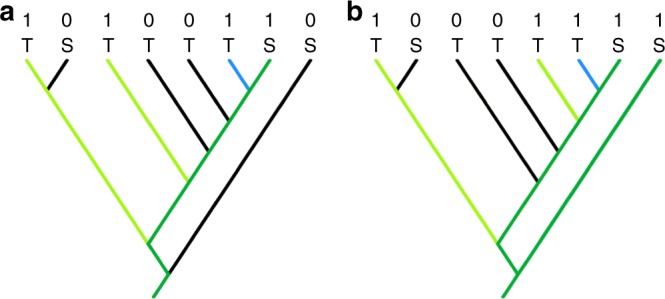
The disconnect between local and global PD priorities. A hypothetical phylogenetic tree of corals showing species presence/absence and PD conservation priorities in two hypothetical ecosystems: **a** reef A, **b** reef B. EDGE does not simply maximise PD, in contrast to Mazel et al.’s^2^ description of the phylogenetic gambit; it uses a range of extinction probabilities for PD priority setting, which we examine here under Mazel et al.’s^2^ focus on local-priority setting. Here, for simplicity, we maximise branch lengths, in assuming that T denotes threatened species with probability of extinction of 1 in the absence of conservation action, and S denotes secure species with probability of extinction of 0. For reefs A and B, 1 and 0 designate presence/absence of species. For each reef, dark green shows already-secure PD associated with secure species. Conservation priorities are determined under a hypothetical budget allowing selection of 2 out of the 3 threatened species (shown in light green) in order to produce a maximum gain in local secured PD. As a result, the species with the blue terminal branch is not selected for conservation in either reef. In this example, we assume that this species is only found in these two reefs; thus, such a narrow ecosystem-level priority setting approach actually allows this species, and its terminal branch, to be lost globally

We then explored the potential magnitude of this disconnect between local and global PD perspectives using information on *Acropora* corals^14^. Under Mazel et al.’s^2^ FD focus promoting more localised PD priority-setting, we show there is a real danger that some threatened coral species are consequently never selected in individual ecosystems, allowing global PD/EDGE losses (Fig. 2).

**Fig. 2:**
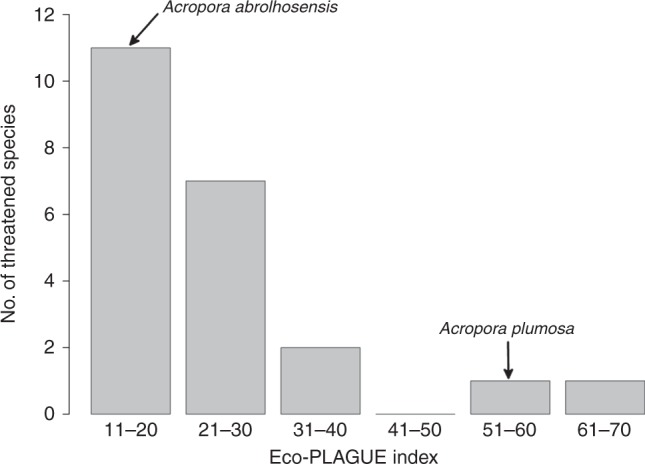
Local priority-setting may allow global coral PD loss. This histogram summarises the extent to which local (within-ecosystem) priority setting for 94 threatened *Acropora* corals allows possible consequent global losses. Local priority-setting explored a range of nominated budgets (for all 141 ecosystems, the budget is the number of species that can be selected for conservation in each ecosystem). For each species, we identified the minimum budget required for it to be selected in at least one ecosystem. This minimum-budget defines the Eco-PLAGUE index, reflecting how much Ecosystem Prioritizing Locally Allows Globally Unexpected Extinction. A larger Eco-PLAGUE index value indicates a greater possibility of global loss of the species, because avoiding loss requires a higher local budget to include this species as part of those selected to be conserved. Many (72) of the 94 threatened species have low (<10) Eco-PLAGUE values and thus would be selected multiple times in individual ecosystems, assuring their conservation. However, the histogram illustrates 22 species with larger Eco-PLAGUE values, showing the total number of these threatened species (*y*-axis) that each require a minimum local budget (Eco-PLAGUE value; *x*-axis) to ensure that the species is selected in at least one ecosystem, so avoiding global loss. For example, *Acropora plumosa* has a very large Eco-PLAGUE index of 57, meaning that it would be less likely to be selected under a local priority-setting approach, and hence more likely to be lost globally, compared to *Acropora abrolhosensis* which has a lower Eco-PLAGUE index of 16

We conclude that Mazel et al.’s^2^ study misrepresents the feature-diversity rationale for PD conservation, and unjustifiably raises doubts about PD applicability in conservation programmes, potentially undermining the growing adoption of PD approaches. Reflecting their exclusive focus on functional traits, their introductory reference to the biodiversity crisis highlights loss of ecosystem functions but neglects loss of global option values. Yet option values were the earliest core values of biotic diversity seen as threatened by the extinction crisis^3^. The Mazel et al.^2^ study is another example of an all-too-common problem in biodiversity science, where an unwarranted exclusive focus on ecosystem functions/services typically implies a critical neglect of global biodiversity values^15^.

## Methods

To illustrate how localised PD priority-setting allows global PD losses, we created a hypothetical phylogeny for eight species, each designated as threatened or secure (extinction probabilities of 1 and 0, respectively), and occurring in one or both of two local ecosystems. PD-based priority-setting within each ecosystem assumed a limited conservation budget allowing selection of the two threatened species that maximise gain in secured PD. Subsequently, global PD loss was indicated by the unsecured branches arising when species were not selected in either ecosystem.

We explored this problem using existing information on *Acropora* corals phylogeny, distribution, and conservation status^14^. The 141 reef ecoregions formed our local reef ecosystems. Securing threatened species provided PD gains, approximated by phylogenetic terminal branch lengths. Local PD priority-setting selected threatened species in order of the magnitude of these gains, up to a nominated budget number of species, applied within each ecosystem. We tabulated: (1) for each nominated budget, species that were not selected in any ecosystem; (2) the minimum budget (applied within all ecosystems) required for each species to be selected in at least one ecosystem, avoiding global loss.

## Data Availability

The *Acropora* corals data are available at Dryad Digital Repository with the identifier 10.5061/dryad.178n3^14^, and other relevant data are available from the authors.
